# Supplementary material for: Are carbon emissions trading and green financial instruments synergistic? -Comprehensive quantitative research based on content analysis
Source: PLoS One. 2024 Mar 7;19(3):e0298601. doi: 10.1371/journal.pone.0298601 (PMC10919601; doi:10.1371/journal.pone.0298601)
Supplement: S1 Data — (ZIP) [file pone.0298601.s001.zip › Data/地级市Z_F工作报告.zip!/╔┬╬≈/░▓┐╡╩╨/└┤╫╘╣1⁄2╓┌║┼í╛┬φ┐╦╩2╛▌═°í┐_2012.docx]

各位代表：

　　现在，我代表市人民政府向大会作工作报告，请予审议。并请政协委员和其他列席人员提出意见。

　　一、关于2011年工作回顾

　　过去的一年，我们在中共安康市委的坚强领导下，在市人大、市政协的监督支持下，坚持以科学发展观为指导，紧紧围绕循环发展、富民强市这一主题，依靠全市人民的团结奋斗，经受住了复杂发展环境和强秋淋天气的考验，圆满完成了市三届人大一次会议确定的年度目标任务，实现了“十二五”发展的良好开局。

　　（一）经济总量快速增长。全年完成生产总值407.17亿元，增长15.5%。全社会固定资产投资304.5亿元,增长29.2%,其中固定资产投资（不含农户投资）234.68亿元,增长30.8%；财政总收入43.5亿元,增长22.2%，其中地方一般预算收入17.3亿元,增长31.1%；全社会消费品零售总额129.32亿元，增长17%；城镇居民人均可支配收入17365元，增长18.6%，农民人均纯收入5009元,增长26%。三次产业结构调整为17.7: 45.0:37.3，二产占比首次超过三产，产业结构发生了历史性变化，向工业化迈出坚实步伐。主要经济指标增速继续位居全省中上水平，生产总值增速全省第三，规模工业增加值和城镇居民人均可支配收入增速全省第一，保持了跟进全省发展步伐的良好态势。

　　（二）新型工业增速强劲。实现规模以上工业产值 299.3亿元，增长49.1%，增加值113.35亿元，增长29%，拉动经济增长6.4个百分点，工业化率提高4.9个百分点。谋划跟踪几年的华电陕煤安康火电、天沐金属镍两个投资过百亿重大工业项目开工建设。陕煤富硒矿泉水、汉阴秦凤电动车、岚皋碳化硅、汉滨众辰机芯、平利凯瑞斯服装等86个重点工业项目建成投产。新增中科纳米、永宏丝业、新农机械等42户产值过亿元企业，新增永丰特种纱、奥邦锻造、居金塬矿业等52户规模企业。与省国土资源厅建立地质资源联合勘探机制，重点矿产勘查取得实效。安康高新区和县域工业园区完成基础设施投资27.5亿元，入园企业达到620户，高新区生产总值增长34.6%，县域工业增长27.5%。呈现出竞相发展的生动局面。

　　（三）投资拉动成效显著。全市在建项目1085个，其中新开工项目709个，投资亿元以上的项目25个。安康至陕川界、安康至白河高速公路建成通车，安平高速开工建设，西康铁路复线、安岚二级公路、安康至白河高等级航道、紫阳两座汉江大桥等重点项目加快推进。完成县乡公路改造300公里、通村水泥路258公里，建成农村公路示范路段3000公里。气化安康主管网工程完工，建成汉阴、白河电气化县，东坝防洪工程试验段、“引汉济渭”工程、洞河水库、白河县城防洪等重点水利项目开工建设。民间投资活跃，投资额达到140亿元，增长42%。金融机构新增贷款47亿元，增长22.3%。投资对经济增长的贡献率保持在60%以上。

　　（四）农村经济活力增强。编制实施现代农业示范园区体系建设规划，启动建设45个现代农业示范园区，阳晨现代农业示范园创建为省级示范园区。粮油总产92.7万吨，连续八年增产丰收。生猪饲养量达到518万头。新增设施蔬菜面积2.09万亩，蔬菜产量113.8万吨，增长5%。发展网箱养鱼2.5万口，养殖大鲵11万尾，水产品产量2.8万吨，增长84.5%。茶叶、蚕桑、魔芋、烤烟、食用菌、干鲜果等特色产业基地稳步发展，富硒品牌建设扎实推进。农村劳动力转移就业68万人，劳务经济收入89亿元。编制实施重点水利项目建设十年规划，新修农田10.4万亩。造林绿化62.4万亩，其中经济林39.9万亩，治理水土流失面积960平方公里。启动实施新一轮开发式扶贫，412个新农村示范村、45个整村推进连片开发村和第二轮100个特困村建设取得新成效。完成第一轮大中型水库移民后期扶持任务。

　　（五）生态旅游日益活跃。深入推进国家优秀旅游目的地创建。加快“一山一湖一城”核心景区建设，建成大南宫山景区南线旅游公路、服务区和四星级酒店。瀛湖招商开发取得进展，4A级景区创建通过省级验收。宁陕、石泉、平利、汉滨四大旅游板块特色鲜明。宁陕休闲养生度假旅游项目呈现集群开发态势，培育出以皇冠、后柳、县河、长安等为代表的一批重点旅游村镇。借助安川高速通车机遇，瀛湖水上旅游、紫阳民俗旅游蓬勃兴起。“把安康带回家”大型宣传活动成效明显。成功举办第二届中国陕西茶叶节、汉江龙舟文化节、“春来早”旅游黄金周、汉阴油菜花节等节庆活动。参展西安世园会获五项大奖，进一步扩大了安康生态旅游的影响力和知名度。全年接待游客1561.9万人次，实现综合收入63.4亿元，分别增长28.2%和27.5%。

　　（六）城镇建设步伐加快。省政府批准我市中心城市总体修编规划，并获省级优秀城乡规划设计一等奖。编制实施安康高新区、月河生态城和城市重点片区控制性详规。瀛湖干道、江北桥头交通组织工程、香溪洞景区道路改造、南环干道B标段、东内环路等项目和一批停车场、市场和城市管网配套工程建成投入使用。启动建设恒口统筹城乡一体化综合配套改革试验区，以月河川道为重点的“一体两翼”城镇带加快形成。以设施配套、扩大容量为重点，有力推进县城、重点集镇和新型社区建设。进一步完善促进农村居民进城的政策措施，全年农村居民进城落户9.5万人。全面完成“7.18”灾害3.08万户倒房重建和5.5万户危房加固任务。双创工作深入推进，省政府授予我市省级卫生城市荣誉，实现省级卫生县城全覆盖。全面启动创建国家卫生城市和国家园林城市。省政府命名我市为省级园林城市，命名平利、旬阳、岚皋、宁陕为省级园林县城。汉阴、宁陕通过国家卫生县城省级考核鉴定。

　　（七）民生工程扎实推进。完成民生工程投资50亿元。实施规模空前的避灾扶贫搬迁安置工程，开工建设集中安置点295个，启动建房29150户116812人，92771名群众搬出山地灾害威胁区。开工建设保障性住房39900套，完成省定任务的114.7%，竣工18667套，1.2万户住房困难群众喜迁新居。石泉县被评为全省保障性住房综合管理工作“十佳县”。提前一年实现城乡居民基本养老保险制度全覆盖，当年参保141.8万人，全市60岁以上老人全部享受到养老保险待遇。城镇职工基本医疗保险实现市级统筹，新农合政策范围内住院费用报销比例提高到70%。新增城镇就业1.5万人,大学生就业3198人，免费技能培训2.8万人，城镇登记失业率3.24%。解决27万人安全饮水问题，8.3万人实现脱贫。建成万村千乡市场工程249个、乡镇超市11个。完成省上下达的主要污染物减排和节能降耗任务，城市空气二级和好于二级天数362天，汉江出境断面水质保持国家地表水II类标准。残联、慈善协会、红十字会充分发挥扶弱济困作用，各条战线、各个方面在推动安康发展与社会进步中都作出了积极贡献。

　　（八）社会事业全面发展。宁陕县成为全省第二个双高双普县，率先在全国贫困地区实现15年免费教育。镇坪县实施高中免费教育，岚皋县整体推进学前教育，安康中学创建为我市第一所省级示范高中。安康职业教育集团组建成立。高考文理二本上线7168人，上线率居全省第二。新建和改扩建幼儿园63所，新增入园幼儿1.4万人。中心城区一批中小学改扩建项目建成投用，新改扩建校舍34.6万平方米。市儿童医院开工建设，实施127个农村医疗卫生服务体系建设项目。完成科技项目49项，科普工作、技术推广和科学研究取得新成绩。人口自然增长率2.87‰，低生育水平持续保持稳定。全国首部实名实景电影《郎在对门唱山歌》在上海电影节荣获五项大奖。《大破天门阵》和《白河水色》获省第六届艺术节19项大奖。振兴汉剧工程扎实启动。建成乡镇综合文化站78个、农家书屋1993个。整合成立市体育运动中心。坚持第一要务抓发展，第一责任保稳定，强力推进平安安康建设，建立健全信访维稳工作机制，加快完善社会治安防控体系，继续加强社会治安综合治理，深入开展严打斗争，大力整治社会治安重点地区，集中解决影响社会稳定的突出问题，清网行动成效显著，全市公众安全感达到95%以上，位居全省第一。突出领导干部接访下访、积案化解、机制创新工作重点，积极创建信访三无县，全面排查化解矛盾纠纷，集中办结一批重大信访案件，实现“一控双降”目标，最大限度地增加了和谐因素，最大限度地减少了不和谐因素，确保了社会大局的持续稳定。重大安全隐患整改率95%以上，安全生产形势稳定。科学应对了六次强降雨过程和强秋淋天气影响，确保了人民群众生命财产安全。

　　（九）改革开放继续深化。全面完成市、县区政府机构改革和撤乡并镇任务，行政区划调整为161个镇办，设置重点镇22个。完成工商、质监下划市县区管理体制改革。调整理顺住房公积金管理、食品安全管理体制。完成市直文化体制改革。全市基层医疗机构基本药物制度实现全覆盖，宁陕、平利县级公立医院改革和县镇卫生一体化改革试点加快推进。农村土地经营权有序流转10.1万亩，启动集体林权制度配套改革，流转林地12.8万亩。公共资产管理改革取得新成效，实现资产收益3.6亿元。制定实施加快金融业发展的指导意见，引进长安银行在安康设立分支机构。新成立小额贷款公司5家，新增典当企业3户。以产业项目为重点的招商引资成效显著，全年引进到位资金208亿元，增长1倍，引进宝钢、中信、三一、紫金等全国500强企业投资安康，新增进出口经营权企业10户，开放型经济发展迈出新步伐。

　　（十）政府建设不断加强。认真贯彻落实市委的决策部署，自觉接受市人大及其常委会的依法监督和市政协的民主监督，完善定期报告工作和联席沟通制度，建立规范性文件报备审查制度。办理人大代表意见建议170件，政协委员提案308件，办结率100%。坚持依法行政，完善科学民主决策机制，推进法治政府建设。以狠抓落实工作年为抓手，建立完善重点任务分解制度和目标责任制，实行月检查、季通报、年评比制度，政府执行力明显提高。扎实抓好政府系统党风廉政建设，深入推进政务公开，加强政风行风评议。加大投资环境专项治理力度，完善重点项目派驻监察室制度，支持监察、审计等部门依法独立履行监督职责。加强基层便民服务体系建设。密切与社会各界的联系沟通，接受新闻舆论、社会公众监督，不断改进政府工作，忠实履行为民服务宗旨。

　　各位代表！过去的一年，我们积极适应宏观政策调整变化，在推动安康发展的实践中，坚持基础先行、交通为首，着力改善加快发展的基础设施条件，使安康在开放发展中紧跟全省步伐；坚持产业兴市、工业引领，紧紧扭住经济建设这个中心，加快构建以新型工业为核心的循环产业体系，有力推动产业结构调整和发展方式转变；坚持招商引资、项目第一，以招商引资聚集要素，以项目投资拉动增长，不断增强经济发展的活力和内生动力；坚持生态优先、循环发展，树立绿色、低碳、环保理念，扎实推进生态文明建设，走出一条资源与环境协调的可持续发展之路；坚持真抓实干、狠抓落实，强化目标责任考核和效能建设，以干事创业的实际行动把加快安康发展的伟大事业不断推向前进。

　　各位代表，过去的一年，我们取得的发展成就令人鼓舞。这是市委正确领导的结果，是市人大、市政协监督支持的结果，是全市干部群众团结奋斗的结果。在此，我代表市人民政府，向全体人大代表、政协委员，向奋战在各行各业、各条战线上的工人、农民、知识分子和干部职工，向各民主党派、工商联、人民团体、各界人士和离退休老同志，向驻安人民解放军、武警官兵、政法干警和复转军人，向所有关心支持安康建设的各界朋友，致以崇高的敬意和衷心的感谢！

　　在充分肯定成绩的同时，我们也要清醒地看到，我市对外开放水平依然较低，基础设施建设仍然滞后，投资环境不优，市场化进程缓慢，经济外向度不高；工业基础依然薄弱，尚处于工业化初级阶段，经济总量小，产业层次低，缺乏大企业、大产业、大品牌支撑；发展动力依然不足，城镇产业支撑和承载能力较弱，资本市场发育不成熟，民间资本不活跃，内生动力不强；群众收入依然偏低，深山区群众生活还比较困难。同时，一些地方和部门思想不够解放，谋事业、抓发展的能力不强，工作落实力度不大。这些问题必须在今后的工作中努力加以解决。

　　二、关于2012年的任务和重点

　　今年是全面贯彻落实市三次党代会精神的启首之年，也是“十二五”发展承上启下的重要一年。既充满机遇，又极具挑战。世界经济形势复杂多变，仍在缓慢恢复和衰退恶化间徘徊。国内经济面临诸多不确定因素，经济存在下行风险，物价上涨压力较大，节能减排形势更趋严峻。兄弟市区争先进位、竞相发展的态势，也给我市紧跟全省发展步伐带来更大考验。同时，挑战中蕴含着诸多新的发展机遇，机遇大于挑战。一是国家突出稳中求进总基调，强化有扶有控宏观政策，在财政方面更加注重向民生、三农等领域和欠发达地区倾斜；在税收方面实行结构性减税、支持小型微型企业发展；在信贷方面支持在建续建重点项目和保障房建设；增加符合产业政策、有市场需求的企业特别是中小企业的投入。国家继续把实施西部大开发战略放在优先位置，促进东中部发达地区的产业转移，加快建设关天、成渝、北部湾和西三角经济区，为我市项目建设、招商引资、投资增长、产业转型创造了有利条件；二是国家大力实施新一轮扶贫开发，下决心解决我市在内的秦巴山区等11个集中连片特困地区的发展问题，在转移支付、重点工程、项目资金、公共服务、产业布局等方面给予特殊政策支持，必将有力推动我市特色产业发展，增强造血功能，缩小发展差距；三是省委、省政府加快实施陕南循环发展，全面落实推进陕南循环发展的若干意见，把新型材料产业发展规划和安康高新区、月河川道现代农业产业基地、恒口统筹城乡发展综合配套改革试验区纳入省级战略层面，大力支持我市重大民生工程与基础设施建设，为我市推进循环发展注入了强大动力。

　　今年政府工作的总体要求是：坚持以科学发展观为指导，全面贯彻落实省“两会”和市三次党代会精神，按照跟进全省发展步伐的要求，以循环发展、富民强市为主题，以转变发展方式为主线，以保障和改善民生为出发点和落脚点，加快构建以新型工业为重点的循环产业体系，突出抓好以产业支撑为重点的城镇化建设，大力推进以农业园区为重点的农业现代化进程，着力改善基础设施条件，继续深化改革开放，更加重视招商引资，加强和创新社会管理，推动文化发展繁荣，为全面建设美好安康努力奋斗。

　　按照市委三届一次全会的安排部署，综合考虑各方面因素，今年经济社会发展主要指标建议为：生产总值490亿元，增长13.5%以上；全社会固定资产投资380亿元，增长25%；财政总收入50亿元，增长17%，一般预算收入20亿元，增长18%；社会消费品零售总额增长17%；城镇居民人均可支配收入增长16%，农民人均纯收入增长18%；城镇登记失业率控制在4.5%以内；居民消费价格总水平涨幅控制在4%左右；人口自然增长率控制在5‰以内；完成省定主要污染物减排和节能降耗任务，汉江出境断面水质保持国家地表水II类标准。

　　实现今年经济社会发展目标，全面建设美好安康，必须把握全局，突出重点，扎实做好十个方面的工作。

　　（一）坚持工业强市，做大新型工业，扶持发展劳动密集型产业，推动工业化进入新阶段。

　　加快发展循环型工业。坚持大企业引领、大项目支撑、集群化发展、园区化承载，集中力量抓好重大工业翻身项目建设。全年完成工业投资100亿元以上，新增35户规模企业，保持工业增加值24%以上的增速。新型材料工业要重点抓好天沐金属镍、陕煤石煤型钒矿、宝钢铌钽、白银铅锌、华银钒氮合金、有色电解锰、吉宇型钢、平利LED光电、石泉重型锻造、岚皋耐火材料等项目；清洁能源工业要重点实施华电陕煤安康火电厂、汉滨延长甲醇汽油、旬阳和白河水电站、镇坪南江河梯级开发、宁陕和平利凯迪生物质能源等项目；富硒食品要重点促进富硒矿泉水、富硒茶饮、富硒粮油、富硒肉制品加工、富硒魔芋食品开发等骨干企业扩大产能，打造“中国硒谷”品牌；装备制造工业要重点加快白河俊达专用车和力神商用车、旬阳球磨铸件、三一工程机械再制造、汉阴阳光太阳能、汉滨智能电视和教学设备制造等项目建设。继续做强生物医药、安康丝绸等传统产业，扎实抓好石泉天汇和白河方宇皂素、汉滨双稀等项目，促进宝业丝绸、巴山丝绢、石泉捻丝等企业发展。加快发展电子信息、环保节能等新兴工业。加快高新区和县域工业园区基础建设，进一步增强园区经济活力。

　　扶持发展劳动密集型产业。发展劳动密集型产业是发展实体经济、扩大城乡就业、促进农民增收、促进社会和谐的有效途径。紧紧抓住东中部地区产业加快转移机遇，充分发挥我市的资源禀赋和劳动力充裕优势，大力引进关联度高、产业链条长的投资项目，加快发展劳动密集型产业及配套企业。劳动密集型产业效益相对较低，对劳动力成本依赖程度高，要从社会保障、土地供应、财税金融、就业奖励等方面给予优惠扶持。

　　重视发展中小型企业。加大技术改造和品牌建设力度，实行小额贷款、税费减免等扶持措施，促进企业转型升级，培育发展一批“专精特新”中小企业。把科技创新工程与企业转型升级紧密结合，全年组织实施10个科技产业化项目、20个重大科技创新项目，扶持科技型中小企业10家，打造高新技术和科技小巨人企业。大力发展非公有制经济，使非公有制经济占比达到49%。

　　（二）加强城乡统筹，强化产业支撑，提升综合承载能力，力促城镇化迈上新台阶。

　　加快构建市域城镇体系。按照“做美城市、做强县城、做大集镇、做好社区”的陕南城镇化发展思路和“三个一百万”的人口布局，明确城镇发展的总体目标、区域布局和功能定位，加快形成以中心城市为核心，以县城和重点镇为依托，以集镇和新型社区为补充的市域城镇体系。

　　强化产业支撑。产业发展是城镇化的持久动力和主要内容。坚持城镇产业建设与基础设施“两条腿”走路、同步发展。加快推进人口向城镇集中、产业向园区集中、土地向适度规模经营集中。引进和建设一批劳动密集型企业，扩大农民就近转移就业规模。推进生产要素向城镇聚集，提高城镇综合承载和辐射带动能力，建成一批工业强镇、旅游大镇、商贸重镇、文化名镇，形成产业聚集区和城镇建设互动发展、产业化和城镇化协调推进的新格局。

　　加快统筹城乡示范区建设。坚持规划引领，编制和实施城乡一体化发展规划、月河川道总体发展规划。按照15万人口、15平方公里新兴小城市的建设目标，加快推进恒口统筹城乡一体化综合配套改革试验区建设，争创国家发展改革试点示范镇。加快形成以中心城市、汉阴和石泉县城、恒口新区和池河集镇为支点的月河川道城镇带。继续抓好石泉杨柳、宁陕皇冠、平利长安、旬阳白柳、白河天宝、岚皋花里等一批统筹城乡发展示范区建设。

　　促进农村居民进城落户。全面落实促进农村居民进城落户的一系列政策措施，彻底打破农民进城的制度障碍。结合避灾扶贫搬迁、保障性住房、重点镇、新型社区、集镇新村建设，解决好进城农民就业、社保、住房、子女入学等实际问题，让进城农民尽快融入城市。优先解决在城市出生的非城镇户籍青少年落户。确保全年实现8万农村居民进城落户。

　　（三）突出市场引领，做优现代农业，推动农业结构调整，开辟农民增收新渠道。

　　加快现代农业示范园区建设。以龙头企业为带动，以“一县一业”为重点，突出抓好以猪、沼、菜（果）循环为主要生产模式的56个现代农业示范园区建设，启动实施43个一县一产业重点项目，加快月河川道现代农业产业基地建设，大力发展生猪养殖、设施蔬菜、水产养殖、特色瓜果、绿化苗木等高效农业。全年出栏商品猪300万头，新增设施蔬菜2万亩，新增大水面养殖2万亩、网箱养鱼1.5万口，新建高效茶园3万亩。稳定粮食播种面积，确保全年粮食产量80万吨以上。不断提高蚕桑、魔芋、烤烟、中药材、食用菌、干鲜果、木本油料等特色产业质量和效益。大规模开展农民技能培训，稳定60万人的劳务转移就业规模。建设一批规范化农业专业合作社，提高农民进入市场的组织化程度。大力发展新型流通业态，加快农产品批发市场建设，建立与现代农业和大市场相适应的加工、储藏、销售、信息体系。促进农业专业化、标准化、规模化、集约化，提高农业综合生产能力、抗风险能力和市场竞争能力。

　　发展山林经济。安康“八山一水一分田”，发展山林经济的潜力很大。要运用现代企业经营理念和工业生产组织方式，培育市场主体，壮大龙头企业，延伸产业链条，注重品牌建设，提升产品附加值，推动山林经济由种养型向加工和市场销售型转变，走规模化经营、产业化发展路子。要分区规划、试点先行、示范带动，推动经济林、生态林、观赏林和林间养殖、林下种植、森林旅游同步发展。

　　全面落实强农惠农政策。继续加大“三农”投入。足额兑现粮食、生猪、农资、良种、退耕还林、水库移民、农机具购置等补助补贴。扩大农业保险覆盖面。加强农民负担监管，规范村级公益事业“一事一议”奖补制度。严格土地管理制度，加强土地整理复垦，实现耕地占补平衡。加快推进开发式扶贫，以101万贫困人口为对象，1099个低收入村、220个片区为扶贫开发主战场，全年投入扶贫资金25亿元，启动建设50个连片开发项目村，继续抓好100个特困村和412个新农村建设，培训农民25万人次以上，完成8万人脱贫任务。实施238个大中型水库移民后期扶持项目，改善26.7万移民群众生产生活条件。

　　（四）加快发展旅游产业，完善配套设施和服务，着力扩大消费需求，打造经济增长新亮点。

　　做强生态旅游。生态旅游是安康实现后赶超的战略性支柱产业，要把生态资源作为一种资本来经营。按照“汉水秦巴秀、自在安康游”的形象定位，以山水为基础，以人文为灵魂，以项目为支撑，全力打造国内一流生态旅游目的地和山水休闲度假胜地。提升“一山一湖一城”标志性核心景区建设水平，深度开发宁陕、石泉、平利、汉滨四大特色旅游板块。加快完善配套服务设施，加大宣传促销力度，提高安康生态旅游知名度和影响力。深入推进岚皋旅游综合改革试点县工作。尽快完成大南宫山景区开发，加快宗教服务区、地质公园和千层河湿地、神河源狩猎场建设。按照大片区、大思路、大规划的要求，整合旅游资源，调整管理体制，引进大企业推进瀛湖大开发，尽快建成国家4A级景区。全面实施旅游整合提升规划，加快香溪洞、朝阳沟、上坝河、深山老林、冰晶顶、天书峡、汉江明珠等重点景区建设，积极开发鬼谷岭、红石河、凤凰山、双龙溶洞等景区。突出30个重点旅游村镇、600个经营示范户的特色化、标准化建设，打造一批旅游名镇、生态小镇、休闲乡村、特色街区。充分挖掘特色资源和文化内涵，促进汉水文化与生态旅游的互动融合。精心办好第十二届汉江龙舟节、美好安康欢乐游、油菜花节、茶文化节等系列节庆促销活动。密切与省内外大型旅游企业和知名景区的合作，开发与西安、重庆、成都、十堰等周边城市的精品线路、旅游环线。全年接待游客1800万人次。

　　促进三产服务业发展。把三产消费作为扩大内需的重点，拓宽和开发消费领域，促进消费结构升级。新增限额以上商贸企业20户。启动建设西坝片区、高新区和月河生态城五星级酒店，提升宾馆餐饮服务业整体水平。加快发展交通运输、文体休闲、物业家政、物流配送等服务业，培育汽车、旅游、文化、健身等消费热点。加强城乡市场体系建设，建成白河国美物流、安康开亮农产品、旬阳现代物流园区、紫阳康宏仓储物流、宝业农贸市场、大南门净菜市场和康兴物流。继续抓好家电下乡、万村千乡和菜篮子工程，搞好农超对接、农产品冷链物流、蔬菜进社区等项目建设。采取有效措施，促进房地产业健康持续发展，多渠道增加住房供应。深入开展诚信安康、好客安康、美食安康创建活动，营造良好的消费环境。

　　（五）坚持交通先行，大力抓好水利和生态建设，继续完善电力通信等配套设施，实现基础条件新改善。

　　加快交通基础设施建设。加快安平高速建设进度，确保十天高速白河至陕鄂界、安岚二级公路、渔紫公路改造、紫阳两座汉江大桥建成通车。开工建设富强机场。加快推进西康铁路复线、西成客专、阳安铁路复线和恒口铁路货运中心建设。实施白河、石泉、岚皋、镇坪县城过境段和平镇、石紫公路等一批干线改造工程，加快白河至安康164公里国家高等级航道建设。开工建设旬阳小河至甘溪、石泉两河至宁陕二级公路。新改建县乡公路200公里,新建通村公路200公里，实施1万公里通村示范路创建和1万公里联户路建设“双万工程”,提高县际、村际道路畅通能力。力争平利至镇坪、安康至岚皋至城口列入国家高速公路网规划，石泉至宁陕、宁陕至丹凤高速公路连接线列入前期工作计划，安张衡铁路列入国家铁路中长期规划。

　　掀起水利建设高潮。以3950个项目为重点，组织实施水利建设十年项目规划。全面加快东坝防洪工程建设，实施西坝二期加固工程。加快实施引汉济渭、洞河水库等骨干水源项目，启动建设汉江综合治理工程，抓好月河补水、双营水库等项目前期工作。加快推进白河县城、旬阳县城和蜀河集镇、汉滨张滩镇和石泉杨柳新区等防洪保安和10个中小河流防洪治理工程，完成15座病险水库维修加固任务。新增基本农田6万亩，新增节水灌溉2万亩,继续抓好县城自备水源工程建设,解决农村20万人的安全饮水问题。治理水土流失面积1000平方公里。

　　加强生态环境保护。山川秀美是全市人民的福祉所在。一江清水不可复制，万重青山难以再造。加快实施汉丹江流域污染防治和水土保持、天然林保护、长江防护林、汉江绿化、千里绿色长廊等重点生态工程。落实节能减排降耗措施，坚持“三同时”和环境影响评价制度，加强工业污染、农村面源污染综合治理。创建1至3个省级生态县，深入开展生态镇、生态村创建工作。建成县城污水处理厂和垃圾无害化处理场。3家以上企业通过省级清洁生产验收。城市空气质量二级和好于二级天数350天以上。

　　推进公共设施建设。加快建设安康至重庆和湖北点对网特高压电力输送通道，实施旬阳330千伏、7个110千伏输变电和城乡电网改造等一批电网工程。争取开展重点企业与发电企业的直接交易试点。实施一批城乡通信基础设施项目，促进三网融合。完成汉中至安康天然气输气管网站配套设施建设，实现气化安康目标。

　　（六）优化投资环境，以承接产业转移为重点，加大招商引资力度，促进开放型经济新突破。

　　强力推进招商引资。坚持招商引资“一把手”负责制，夯实各县区、各部门的招商任务。围绕优势资源开发和主导产业建设，瞄准省内外大企业，有针对性地策划包装一批好项目，在北京、上海、江苏、广东等地举办招商活动，加强与北京石景山区、江苏常州等发达地区的经贸协作。重视发展与关天、成渝、江汉等周边经济圈关联度高的配套产业，主动承接长三角、珠三角、环渤海等发达地区的产业转移。各县区要主动走出去，加强项目推介、招商洽谈，推动与东部市县进行点对点、城对城对接和承接产业链整体转移。全年引进到位资金200亿元以上。大力发展对外贸易，建设安康陆路货运口岸，把我市茧丝和中药材列入全省外贸出口基地。

　　营造良好投资环境。进一步减少和规范行政审批，清理精简收费项目。强化政府绩效管理和行政问责制度，完善部门联审、限时办结、全程服务、跟踪落实的工作机制。规范执法行为，健全重点项目、重点企业派驻监察室制度，公开曝光处理有损投资环境的行为。

　　（七）加快民生工程建设，全面发展社会事业，提升公共服务水平，确保社会保障能力新提高。

　　着力保障和改善民生。把促进就业作为经济社会发展的优先目标。坚持以创业带动就业，大力推进全民创业、家庭创业、自主创业。加强职业技能培训，多渠道开发就业岗位，重点解决好高校毕业生、复员退伍军人、进城落户农村居民和就业困难人员的就业问题。全年新增城镇就业1.48万人，大学生就业3200人。继续加快保障性住房建设，全年新开工保障性住房3.37万套，续建2.1万套，健全完善资金筹措、土地供应、质量监管和使用管理工作机制，确保公开公平公正分配。加快完善覆盖城乡居民的社会保障体系，推动养老、医疗等社会保障从制度全覆盖到人群全覆盖。全面落实城乡低保、五保户供养、困难救助等民生政策。启动中心城区老年公寓和社会福利院项目，引入市场机制推动机构养老和社区养老发展。保持物价水平基本稳定，完善物价涨幅与低收入群体生活补贴联动机制。进一步扩大新农合、城镇居民基本医疗保险覆盖面，继续实施基本公共医疗卫生服务项目，新改扩建一批县级医院、乡镇卫生院和社区卫生服务中心，培训全科医生150名。扎实抓好国家计划生育优质服务县等创建工作，实施母亲健康、优生促进、计生家庭创业三大工程，稳定低生育水平。启动县级公共体育设施“四个一”工程，加快县级全民健身中心和体育场建设。

　　扎实推进避灾扶贫搬迁工程。让22万户88万群众从根本上摆脱山洪和地质灾害的威胁，是我们尊重自然规律、实现长治久安的治本之策。要结合推进城镇化和新农村建设，坚持科学规划、基础先行和节约集约资源，重视抓好产业发展、基础配套与社会服务管理创新。挖掘现有财力，多方争取资金，让搬迁的速度更快一点、让搬出的人更多一点，力争提前完成搬迁任务。全年完成3.1万户11.4万人的搬迁安置任务。

　　坚持教育优先发展战略。统筹全市教育资源，促进各类教育均衡发展。实施学前教育三年行动计划，新建40所县城标准化和镇办中心幼儿园，学前教育三年入园率达到70%。加快普及高中阶段教育，扩大中心城市和县城高中办学规模，加快白河高中迁建，全市高中阶段入学率达到85%。优先解决城镇学校“班大人多上学难”问题，完成35.6万平方米校舍建设工程。加快宁陕省级教育强县创建，通过平利、白河“双高双普”验收，推进汉阴、镇坪“双高双普”工作。提高义务教育公用经费标准。认真落实省市共建安康学院协议。健全完善助学机制和家庭困难学生资助体系，启动实施农村义务教育学生营养餐计划，解决好留守儿童、农民工随迁子女、残疾少年儿童及孤儿等特殊困难群体上学问题。推进实施人才中长期发展规划，建设高素质人才队伍。

　　推动文化大发展大繁荣。建立完善覆盖城乡、结构合理、功能健全、实用高效的公共文化服务体系。加快安康博物馆、图书馆、影剧院、文庙、西城阁、汉江古会馆群等项目建设。实现行政村农家书屋全覆盖，完成4300个20户以下自然村和74个新通电行政村广播电视村村通工程。深入实施振兴汉剧工程，精心打造汉剧现代大戏，带动民歌、龙舟、茶饮、民俗文化加快发展。做好新一轮安康市志编纂工作。发挥高等院校和文艺团体作用，推动我市科技和文化生产力发展。

　　（八）深化体制改革，破解发展难题，积蓄能量和后劲，增添经济发展新活力。

　　加快投融资平台建设。资本市场发育不健全、建设资金不足是制约安康经济发展的突出问题。要尽快转变依赖经营土地筹集建设资金的方式，更多利用融资平台撬动社会资本，最大限度用足用活银行资金，扩大社会融资总规模。以市国有资本经营、公共资产投资和城市建设投资公司为主体整合成立安康发展投资集团。做大做强高新产业发展公司。引进股份制商业银行在安康设立分支机构。积极发展村镇银行，新增5户小额贷款公司，新增2户融资性担保公司，新增1户典当企业。扩大市财信担保公司注册资本金规模，今年要达到3亿元以上。进一步密切与金融机构的合作，引导资金投向，加强银企对接，扩大信贷规模，提高贷款投放比例。推进使用票据贴现、银团贷款、短期融资券、集合票据等新型金融产品。加快发展保险业。加强社会信用体系建设，建立覆盖全市的征信系统。

　　深入推进重点领域改革。启动新一轮投资体制改革，进一步下放项目审批和核准权限，开展政府投资项目公示试点。以放活经营、规范流转、保护生态、做大产业为核心，加快林权制度配套改革。全面推进十县区县级公立医院改革，在县级医疗机构实施基本药物和药品“三统一”制度。深化文化行政管理体制改革，推进文化事业单位改革，完善经营性文化单位运行体制。推进粮食、供销、公共资产管理和农村信用社改革。稳步开展事业单位清理规范，有序推进教育、科技、卫生、体育等事业单位分类改革。逐步引入市场竞争机制，改革基本公共服务提供方式，满足群众多元化需求。

　　（九）加强城市管理，推进高新区建设，美化绿化江南老城，塑造中心城区新面貌。

　　实施城市北扩战略。目前，江南老城区人口稠密、道路狭窄、拥挤不堪，必须加大中心城市北扩力度。从今年起分期分批实施江南机关单位北迁。严格控制江南主城区开发强度，搬迁腾出的土地主要用于街心公园和市政公共配套设施建设。加快江北新区开发，建成滨江三期、汉江天然浴场等项目，加快四校北迁、儿童医院、汉城商业街、保障性住房等重点工程，开工城东大桥、汉调二黄文化园二期等重点项目，实施一批骨干路网、污水管网、天然气供应、强电入地等市政配套工程。高起点推进月河生态新城开发，启动建设展示中心、新罗路等项目。

　　加大高新区开发力度。突出抓好主导产业、基础设施、科技创新、投融资平台建设，加快建成一批新型材料、富硒食品、生物医药、节能环保和特色服务产业。快速推进金属镍循环产业园建设，确保增速30%以上。建成秦岭大道、汉江路等一批基础设施，加快创业大厦、商业综合体、三大主题公园、市民广场和高新中小学、幼儿园、医院等项目建设。实施创新路、科技路等十条路网工程。开工高新产业孵化园、商业公建工程和居尚花园、傅家河、建民三大社区，带动江北新区开发提速。

　　改造提升江南老城。引进国内知名大型企业财团，加快西坝防洪与旧城改造工程、东坝防洪保安与综合开发工程建设，把西坝片区建成高档商业聚集区，把东坝片区建成大型综合小商品市场，配套发展物流、商住、休闲、旅游等服务业，使之成为中心城区新的经济增长点和展示城市风貌的新窗口。完善江南城区配套功能，建成南环快速干道、南环东路、内环路文化墙、水西门城楼、兴安公园，开工建设新城一期改造、高井路二期和城区骨干道路改造、人防综合利用工程等项目。加快建设一批停车场、市场、环卫等市政设施。启动“数字安康”地理空间框架建设，加快城市建设管理信息化进程。创新中心城市管理体制，推行“大城管”管理模式，强化规划执法监察，加大城市综合整治力度，全面提升城市管理水平。

　　绿化美化中心城区。绿化既是高水平、低投入的城市生态美化形式，也是绿色、循环、富民的经济发展方式。安康具有大规模、高标准实施绿化的条件，要加快绿化步伐，美化城市环境。进一步加大双创力度，今年实现省级园林县城全覆盖，通过国家卫生城市和国家园林城市省级技术调研，力争提前一年实现创建国家园林城市目标。按照“一心两廊多带一圈”的中心城市绿化框架和大中小绿地并举、点线面绿化结合的要求，从街景绿化到庭院绿化、从平面绿化到垂直绿化进行科学合理布局。全面实行党政机关、企事业单位义务植树责任制，广泛开展绿化月、植树周活动，大面积、大规模植树造林。今年春季，突出抓好中心城区、高新区和交通主干道绿化。

　　（十）加强和创新社会管理，强化安全发展理念，完善应急管理体系，开创和谐稳定新局面。

　　提高维稳工作水平。稳定事关全局，稳定压倒一切。今年国家和省上重大政治活动集中，必须把维护稳定放在突出重要位置。切实加强组织领导，完善工作机制，健全工作机构，充实队伍力量，增加经费投入，提高防控能力。深化平安安康建设，加快完善城乡防控体系，实施社会稳定风险评估，强化严打斗争，突出集中整治，依法严厉打击犯罪活动。以群众工作统揽信访工作，坚持和完善领导接访、下访、回访和包案制度，不断完善信访责任体系、考核评价、目标管理和联合调处机制，依法规范信访秩序，深入开展信访“三无”县区创建。及时回应社会关切，主动解决群众合理诉求，最大限度把矛盾解决在基层、化解在萌芽状态。扎实开展“六五”普法，提高全民法制意识，推进法治安康建设。

　　提升防灾减灾能力。以汉江防洪和山地灾害防范为重点，落实预警到户、科学调度、撤离转移等有效措施，深化全民防灾减灾警示教育宣传月活动，进一步完善防抢撤预案和应急管理体系，不断提高灾害防范和应急处置能力，确保人民群众生命财产安全。

　　稳定安全生产形势。牢固树立安全发展理念，认真落实安全生产责任制，加大安全设施投入力度，加快推进企业安全标准化建设，深入开展隐患排查整治行动，严防发生重特大安全事故。加强食品药品安全监管。

　　合力建设和谐社会。最大限度激发社会活力，最大限度增加和谐因素，最大限度减少不和谐因素。积极构建和谐劳动关系，有效维护农民工合法权益。深入开展双拥共建，支持武装警察部队、消防部队建设，加强国防后备力量建设。依法管理宗教事务，重视解决少数民族群众的生产生活困难。发展老龄、残疾人、慈善事业。搞好外事侨务对台工作。支持工会、共青团、妇联、科协、工商联、红十字会等群团组织在经济发展和和谐社会建设中发挥更好的作用。

　　三、关于政府自身建设

　　面对建设美好安康的新任务、新要求，我们必须进一步强化以人为本、执政为民理念，转变政府职能，提高行政效率，忠实履行服务发展、造福人民的神圣职责。

　　第一，坚持依法行政，自觉接受监督，强化民主法治政府建设。围绕建设法治安康，自觉把依法行政贯穿于政府工作全过程，严格按照法定权限和程序行使权力。努力争创首批省级依法行政示范市。坚持科学民主决策，实行专家论证、公众参与、风险评估、新闻发布、决策公示制度，扩大群众知情权、参与权、监督权。强化行政权力监督，坚决贯彻市委的决策部署，自觉接受人大及其常委会的依法监督和政协的民主监督，重视司法监督、舆论监督和社会公众监督。强化政府督查和效能监察，确保行政权力依法、阳光运行。

　　第二，坚持以人为本，保障改善民生，强化为民亲民政府建设。深化政府系统创先争优为民服务活动，把群众的呼声、群众的意愿和群众的诉求作为施政理政的第一信号，把维护人民群众的根本利益作为想问题、办事情、做决策的出发点和落脚点。坚持民生优先，心系群众冷暖，情牵百姓喜忧。更加重视弱势群体生产生活，更加重视群众合理合法诉求，更加重视维护社会公平正义。

　　第三，坚持真抓实干，不做表面文章，强化求真务实政府建设。为政在行，以实则治。牢固树立正确的政绩观，坚决查实情、说实话、出实招、求实效。不讲不实之词，不干不实之事，不贪一时之功，不图一时之名。多做打基础、利长远的工作，让所做工作经得起实践的检验，经得起历史的检验，经得起人民的检验。按照“作风转变年”总体工作部署，深入实际，深入群众，落实首问负责、服务承诺、责任追究等制度，减少非公务应酬，减少文山会海。咬定任务不放松，扑下身子抓落实。

　　第四，坚持艰苦朴素，反对奢侈浪费，强化勤俭节约政府建设。安康还很落后，还有上百万群众处在贫困线以下，无论在任何时候，我们都要始终保持艰苦奋斗的作风。时刻牢记节约光荣、浪费可耻，珍惜民力民财，厉行勤俭节约，狠刹奢侈浪费，降低行政成本。严格执行财经制度和纪律，规范政府采购，从严控制各项行政开支，从严控制行政机关、事业单位楼堂馆所建设，今年做到公车购置、会议经费、公务接待、公款出国出境等经费支出零增长。

　　第五，坚持反腐倡廉，加强权力制约，强化清正廉洁政府建设。风清气正是人民所盼。官廉民之福，官贪民之祸。要大兴清正之风,大倡廉洁之行。坚持廉洁从政、从严治政，认真落实标本兼治、综合治理、惩防并举、注重预防的方针，严格执行党风廉政建设责任制，扎实抓好政府系统廉政建设和反腐败工作。政府工作人员特别是领导干部一定要增强廉政意识，带头落实廉政准则，做到权为民所用、情为民所系、利为民所谋。

　　各位代表！崛起的安康正在破浪前行，美好的安康需要齐心铸就。让我们高举中国特色社会主义伟大旗帜，在中共安康市委的坚强领导下，坚定志在必得的信念，弘扬一往无前的精神，解放思想，开拓进取，以更加优异的成绩迎接党的十八大和省十二次党代会的胜利召开！
